# Case Report: Making a diagnosis of familial renal disease – clinical and patient perspectives

**DOI:** 10.12688/f1000research.11316.1

**Published:** 2017-04-12

**Authors:** Zahra Iqbal, John A. Sayer

**Affiliations:** 1 Renal Services, Newcastle upon Tyne NHS Foundation Trust, Newcastle, NE77DN, UK; 2 Institute of Genetic Medicine, Newcastle University, UK, Newcastle, NE1 3BZ, UK

**Keywords:** focal segmental glomerulosclerosis, genetics, whole exome sequencing, TRPC6, podocytye, proteinuria, ethics

## Abstract

Background: A precise molecular genetic diagnosis has become the gold standard for the correct identification and management of many inherited renal diseases.

Methods: Here we describe a family with familial focal segmental glomerulosclerosis, and include a clinical and patient perspective on the diagnostic workup and relaying of genetic results following whole exome sequencing.

Results: Through next generation sequencing approaches, we identified a pathogenic mutation in *TRPC6*, the underlying cause of the phenotype. The identification of this mutation had important clinical consequences for the family, including allowing a living-unrelated kidney transplant to proceed in the index case. There are also wider ranging social and ethical dilemmas presented when reaching a genetic diagnosis like this one, which are explored here by both physicians and the index case.

Conclusions: Through physician and patient perspectives in a family with inherited renal failure we explore the implications and the magnitude of a molecular genetic diagnosis.

## Introduction

Familial renal disease is a challenging problem, in terms of diagnosis, treatment and ethical decisions. Here we describe a family affected by a familial form of focal segmental glomerulosclerosis (FSGS), which has resulted in end stage renal disease (ESRD) in two family members, with other family members at risk of the same disease. We wished to explore the significance of making a genetic diagnosis of familial ESRD and the impact of such a diagnosis on the index patient and their family. We therefore outline both the clinical and patient perspective of the index patient and her family.

## Clinical case report

**Figure 1.**
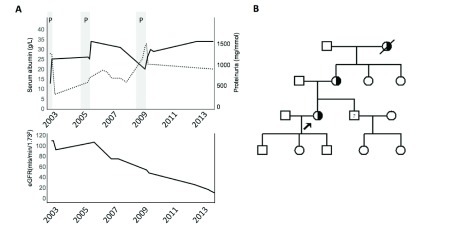
Clinical progression of index case and family pedigree. **A**. Clinical progression of index case over time with serum albumin (solid line), urine protein/creatinine ratio (dashed line) and estimated Glomerular Filtration Rate (eGFR). Pregnancies marked with shaded area (P). **B**. Family tree with index case arrowed. Males are squares, females are circles. Heterozygously affected individuals are semi-shaded. Sibling with proteinuria marked with “?”.

The index case presented in 2003 at the age of 30 years to renal services after her first pregnancy in 2003. She had developed heavy proteinuria and hypoalbuminemia during her pregnancy. After delivery of a healthy son at 40+2 weeks, her proteinuria reduced from a urine protein/creatinine ratio (uPCR) of 1200mg/mmol to 350mg/mmol at 6 months post-partum ( Figure 1A). Her serum creatinine and blood pressure values remained normal during this pregnancy.

A positive family history of renal disease was known ( Figure 1B). The index case’s mother had presented similarly during her first pregnancy at age 30 in 1973. Her renal function steadily declined despite commencement of an ACE inhibitor and she reached ESRD in 2015 at the age of 70 years, and was commenced on peritoneal dialysis. In addition, a maternal grandmother had died in her 60s of “renal disease” but the exact diagnosis was unknown. The index case also had two maternal aunts who are not known to have renal disease. At this stage, no other family members had presented with symptoms consistent with renal disease.

In 2004, due to persistent proteinuria, 10 months after her first pregnancy, the index patient underwent a renal biopsy, which demonstrated FSGS. This was managed conservatively. At the age of 32 years and with careful pre-conception counselling, our patient conceived her second child. Her proteinuria again increased during this pregnancy. A healthy daughter was delivered successfully and post-partum, the proteinuria settled (uPCR = 650mg/mmol). During her third pregnancy aged 36, her proteinuria increased dramatically (uPCR = 1090mg/mmol at 20 weeks gestation and 1340mg/mmol at 33 weeks’ gestation). This was associated with other features of nephrotic syndrome, including serum albumin of 20g/L and estimated Glomerular Filtration Rate (eGFR) declining to 45ml/min/1.73m ^2^ ( Figure 1A). This prompted early delivery of her son at 36+5 weeks at a weight of 2.3kg, who then required a ten day stay at the specialist baby unit.

Following this third and final pregnancy, blood pressure was optimised with a combination of angiotensin receptor blockers and thiazide diuretic within the renal clinic. Despite these measures, her eGFR continued to progressively decline ( Figure 1A) and she received counselling and information regarding the various methods of renal replacement therapy, opting for peritoneal dialysis when required. Her husband offered to be a living-unrelated kidney donor and pre-emptive renal transplantation work-up was commenced.

Given the likelihood of familial FSGS leading to ESRD, based on renal histology and the clinical course of the index case and her mother, genetic studies were initiated, following informed consent from the index case and her mother. Targeted genetic studies excluded mutations in *WT1* and *NPHS2* genes, and this was followed by whole exome sequencing which identified a known pathogenic variant in *TRPC6* (c.2683C>T; p.Arg895Cys) ( Table 1), which segregated from the affected mother. The finding of a genetic mutation causing FSGS meant that the likelihood of recurrence of FSGS in a non-related donor was low and her living-unrelated transplant surgery was expedited. There have been no known recurrences of FSGS after renal transplants in patients with underlying mutations in *TRPC6* ^1, 2^.

**Table 1.** *In silico* analysis of *TRPC6* variant.

Gene Variant	MutationTaster	Evolutionary Conservation	SIFT	ExAC database	PolyPhen-2	dbSNP	Ref
*TRPC6* c.2683C>T p.Arg895Cys	Disease causing	*D. rerio*	Damaging	Not found	Probably Damaging	rs121434394	Reiser *et al.* 2005

In 2014, at age 41, the index case received a pre-emptive living-unrelated renal transplant from her husband, which had immediate graft function. Her transplant function remains excellent with no evidence of recurrent FSGS.

More recently, the index case’s brother was identified as having heavy proteinuria in 2016 at age 40, and is undergoing further investigations ( Figure 1B). The three children of the index patient, who are fit and well, have not yet been tested for the disease causing variant.

## Genetics and underlying mechanism of disease of familial FSGS

The first identification of human *TRPC6* mutations was reported in 2005. Here, a point mutation in *TRPC6* was identified in a family with autosomal dominant focal segmental glomerulosclerosis ^1^. Since then, several other mutations in *TRPC6* have been described. The *TRPC6* p.R895C heterozygous mutation that we report here has been described previously in a large Mexican family ^2^. Here, 9 of 25 family members were affected and presented between the ages of 18 and 46 years, and 6 of the family members reached ESRD. Of these, 2 had received renal transplants with no evidence of recurrent disease. TRPC6 is a non-selective cation channel ^3^ which is expressed in podocytes and glomerular endothelial cells ^2^. TRPC6 channel activity at the slit diaphragm is required for the regulation of podocyte structure and function ^2^. Biophysical analysis of the p.R895C mutant TRPC6 channel showed pathogenic changes in the current-voltage relationship which were suggestive of a gain-of-function ^2^, which *in vivo* would be predicted to increase calcium influx. Interestingly, podocytes express other TRPC channels, including TRPC1, TRPC2 and TRPC5, and an overlap in function may account for the usual adult onset of glomerular disease. Another level of complexity is that TRPC6 may also form heterotetramers with other TRPC channels ^2^. The fact that the p.R895C mutation causes a gain-of-function means that selective TRPC6 inhibitors such as larixyl acetate may represent a pharmacological therapy for this form of FSGS ^4^. More recently, a role for *TRPC6* in renal fibrosis has been identified, which may spur on efforts for the clinical use of TRPC6 inhibition in other progressive renal diseases ^5^.

## Patient perspective

“Even though my mother had a history of renal disease, and I had presented with proteinuria during my first pregnancy, there had been no suggestion made to me, or present in my mind of a possible genetic renal condition. When following my biopsy in 1999, I received a probable diagnosis of familial FSGS, it came as a huge shock, not only to hear I had FSGS, but also the rarer familiar form. Furthermore, knowing you have a rare chronic illness is one thing, but more significantly, I was devastated about what the future might hold for our children.

With this is mind, we began enquiries about how to find out which faulty gene had caused the FSGS and it was decided to undertake genetic tests including sequencing my whole exome. Whilst the result might not help in the short term, it would be useful in terms of being able to test other family members in the future.

I remember the consultant saying that finding the change in the faulty gene was like looking for 1 change in 6 billion pieces of genetic code and the expression ‘needle in a haystack’ was mentioned. Even after such detailed analysis I was told that the results are sometimes inconclusive. Not wanting to miss an opportunity, I flippantly mentioned screening for other faulty genes – by which I mean other non-renal conditions. I did not consider the possibility that our type of familial FSGS may be caused by more than one faulty gene and this is a serious and worrying consideration for patients waiting for the results of any genetic sequencing, notwithstanding the added complications it infers for future research and potential management or cure.

The screening process took a long time (over 6 months) and several clinic appointments passed before we received the results. Fortunately, the investigations were positive and as strange as it sounds, I was pleased to be told that I carried a variant in the *TRPC6* gene. No other faulty genes were identified. My mother was extremely interested in the diagnosis given her condition has been termed “nephrotic syndrome” for thirty years but this was tempered by an ill-founded sense of guilt that she had passed on her condition to her daughter. This is an emotion I can identify with in terms of my own children.

Consequently, the excitement about a positive result naively produced a sense of hope about a potential innovation in the near future, given we had a precise genetic cause. *TRPC6* encodes a calcium channel and based on current understanding the protein is expressed in the podocyte of the kidney, an area currently undergoing a lot of research. Whilst the condition is extremely complex, this form of FSGS may well be a candidate for clinical trials aiming to modify the faulty channel.

As a patient, having something concrete to hold on to, such as the likely cause of our condition, provided some comfort and a sense of empowerment. Receiving the news that the cause of our FSGS was genetic meant it was much less likely for proteinuria to reoccur in a transplant, whereas the risk of recurrence is high in patients with other forms of FSGS. The prospect of immediate kidney rejection is daunting even without the added anxiety of the disease reoccurring and causing rejection, and having this information was an enormous relief for our family. In addition, awareness of this mutation now means that other family members (should the need arise) need only have a blood test rather than a kidney biopsy.

The sting in the tail, in our particular case, is that the pathogenic variant of *TRPC6* remains a very rare cause of familial FSGS, with only a small amount of published reports for doctors to refer to. The rate of deterioration in kidney function has been very different in myself and my mother, whose renal replacement therapy began at 70 years of age. In 2016, my brother presented with proteinuria and mildly raised blood pressure, and is awaiting the results of his genetic tests. He has two children and will no doubt have considered the possibility that they may also be vulnerable. We have all had unique experiences, and this does not make the analysis easier for the nephrologists, or give them the tools to predict future outcomes.

Identifying the variant in *TRPC6* contributing to our form of familial FSGS, does however open up the opportunity to support directed research studies and help further the knowledge about this condition. At this stage, we have decided with the support of the nephrologists, not to test our children, and will do so when the time is right. Yet whilst the threat of this condition hangs over their heads, we continue to fundraise and support renal research in the hope that one day a cure may be found.”

## Discussion and conclusions

Using a clinical case summary and a reflective patient perspective, we provide an example of how a molecular genetic diagnosis in a life threatening inherited renal disease may provide an explanation of the underlying disease process and offer the ability for screening of other family members without the need for invasive tests such as renal biopsy. A genetic diagnosis, by its very nature, also raises issues within the patient and their family members, which may be far reaching. Importantly, a genetic diagnosis often furthers our knowledge of disease phenotypes in rare inherited disorders, and hopefully provides momentum for future research into precision medicine therapies. Engagement of patients and their families in the importance and value of genetic and genomic data for diagnostic, therapeutic and prognostic use should be actively encouraged. Mainstreaming of genomic medicine into medical specialties such as nephrology needs to be embraced by patients and their physicians.

## Consent

Written informed consent was obtained from the patient and family for publication of this case report and any accompanying images and other details that could potentially reveal the family’s identity.
