# Supplementary material for: Case Report: Making a diagnosis of familial renal disease – clinical and patient perspectives
Source: F1000Res. 2017 Apr 12;6:470. [Version 1] doi: 10.12688/f1000research.11316.1 (PMC5443337; doi:10.12688/f1000research.11316.1)
Supplement: Peer review history

# Peer review: Referee response for version 1

**DOI:** 10.5256/f1000research.12212.r22500

**Published:** 2017-05-23

**Authors:** Beata S. Lipska-Ziętkiewicz

**Affiliations:** 1 Department of Biology and Genetics, Medical University of Gdańsk, Gdańsk, Poland

The authors present the insidious course of an infrequent subtype of FSGS, the *TRPC6*-related glomerulopathy, that is an extremely rare renal disorder with ca.100 families diagnosed worldwide. Most likely an individual nephrologist will see no more than one-two such cases during his/her years of practice. The presented paper is an excellent source of information on how to comprehensively handle such patients, not only from medical but also emotional and ethical perspective.  

 The strong points of the work are 1) presenting the recent advances in diagnostics resulting from rapid improvements in genetic sequencing technologies making kidney biopsy (almost) obsolete; 2) including the patients perspective and highlighting their engagement not only in clinical management but also in research .

 The weak point is too superficial presentation of the current standards of preemptive genetic testing in minors for late-onset conditions. I would recommend at least adding a reference to European Society of Human Genetics  position on the issue (for details see: https://www.eshg.org/eshgdocs.0.html) and/or paragraph discussing the ethical, legal, and psychosocial implications of such genetic testing.  

 I would also suggest to modify Figure 1B (family pedigree). Currently it seems to report results of genetic testing in the entire family, but most of the family members were not subject to any genetic testing, the affected grandmother included. Therefore, I recommend that the graph reports on phenotype and not genotype, ie. the individuals with phenotype expressed should be marked as “fully filled-in” while the information re: mutational status is to be provided below where available.

I have read this submission. I believe that I have an appropriate level of expertise to confirm that it is of an acceptable scientific standard.

# Peer review: Referee response for version 1

**DOI:** 10.5256/f1000research.12212.r22625

**Published:** 2017-05-22

**Authors:** Larissa Kerecuk

**Affiliations:** 1 Department of Pediatric Nephrology, Birmingham Children's Hospital, National Health Service Foundation Trust, Birmingham, UK

Excellent case report highlighting an important area of genetic testing being very useful for managing the patient. The report shows the importance of taking a family history in any disease especially renal and exploring this further. Having the patient perspective adds a different angle which is very important for doctors to be aware of as lots of learning can happen from this.

I have read this submission. I believe that I have an appropriate level of expertise to confirm that it is of an acceptable scientific standard.

# Peer review: Referee response for version 1

**DOI:** 10.5256/f1000research.12212.r21845

**Published:** 2017-05-19

**Authors:** Aoife M. Waters

**Affiliations:** 1 UCL Institute of Child Health, University College London, London, UK

Familial cases of rare diseases provide a remarkable opportunity to delineate important biological processes relevant to human disease. Of particular importance, are the implications for future therapeutic strategies for refractory and progressive diseases such as focal segmental glomerulosclerosis. 

 Iqbal and Sayer through their report elegantly present the natural history of TRPC6-associated glomerulopathy arising as a result of the mutation c.2683C>T; p.Arg895Cys which concurs with previous reports of disease manifestation of the same genotype by Reiser et al in 2005. Presentation tends to have an insidious onset in mid-adulthood and despite intervention with ACEIs, patients with this genotype progress towards end-stage renal disease. Functional characterisation of the TRPC6 variant, p.Arg895Cys, utilising electrophysiological studies, revealed the likelihood that this variant represented a gain of function in the encoded mutant protein. Increased intracellular calcium influx as a result of gain of function mutations in TRPC6 have been shown to lead to increased podocyte apoptosis, a common pathogenetic mechanism of FSGS. Therefore,  downstream inhibition of increased TRPC6 activation, represents a therapeutic strategy for patients with TRPC6-associated glomerulopathy. As highlighted by Iqbal and Sayer, without effective treatment for this disease, progression to end-stage renal disease and renal transplantation occurs. For this particular variant, the risk of recurrence post renal transplant is negligible.

 Including the patient perspective is an innovative approach to the case reporting of interesting pedigrees of rare disease. By undertaking such a strategy, Iqbal and Sayer highlight the importance of patient engagement in genomics research by consent for testing of additional affected and unaffected family members to fully characterise the genotype-specific disease manifestations. Furthermore, it provides an answer for the affected patients and provides a rationale for clinical decisions relating to their care.

 Importantly, this report also provided perspective with regards to future testing of prospective asymptomatic younger family members and whether to burden families with advance knowledge of prospective disease susceptibility.

 What this knowledge does offer, of course, is the possibility to influence the disease course prior to manifestation of overt disease symptoms and careful consideration of this prospect should be highlighted at time of consent.

I have read this submission. I believe that I have an appropriate level of expertise to confirm that it is of an acceptable scientific standard.

# Peer review: Referee response for version 1

**DOI:** 10.5256/f1000research.12212.r21847

**Published:** 2017-05-02

**Authors:** Moin A. Saleem

**Affiliations:** 1 Children's Renal Unit and Academic Renal Unit, Bristol Children's Hospital, University of Bristol, Bristol, UK

This is an interesting way of presenting a familial case of renal disease from the patient perspective. The case illustrates how a new diagnosis of monogenic (Mendelian) disease can impact on the family, by firstly clarifying the underlying cause, and being able to discuss in more detail the prognostic and genetic testing implications.

 It also illustrates the powerful impact of the rapid improvements in genetic sequencing technologies, and how these have translated into clinical practice. It is worth emphasising that this will have impact on all clinicians treating renal (and other) diseases, who will need to keep up to date with the current screening technologies and interpretation/limitations of the data generated. In the UK routine whole genome sequencing of patients with rare diseases is being rolled out, so there will be more cases like this being uncovered and requiring counselling.

 One key issue touched upon is the ethical implications of testing younger family members, who may (or may not) develop disease much later in life. The consensus at the moment is to wait until the young person is of an age to make the decision to test independently, unless there are treatment implications of knowing earlier in life. With this particular mutation this is unlikely to be the case, though cases of TRPC6 mutations have been reported to present with proteinuria as early as 6 years of age.

 Another ethical consideration of whole exome or genome sequencing is of finding completely unrelated potentially pathogenic mutations. This needs careful pre-counselling before the test is done.

I have read this submission. I believe that I have an appropriate level of expertise to confirm that it is of an acceptable scientific standard.
